# Ion intercalation, redox, and interfacial mechanisms of MXene-based negative-electrode systems for rechargeable batteries

**DOI:** 10.3389/fchem.2026.1913683

**Published:** 2026-08-14

**Authors:** Bangsheng Yin, Shengjun Ji, Jiawen Tian, Haotian Wu, Wenyue Si

**Affiliations:** College of Mechanical Engineering, Shandong Huayu University of Technology, Dezhou, Shandong, China

**Keywords:** interfacial regulation, ion intercalation, MXene, negative-electrode systems, pseudocapacitance, rechargeable batteries, redox mechanism, surface terminations

## Abstract

MXenes combine metallic conductivity, redox-active transition-metal layers, and chemically tunable surfaces, making them attractive components of negative-electrode systems for lithium-, sodium-, potassium-, zinc-, and multivalent-ion batteries. However, interpreting MXene-based negative-electrode systems only by reversible capacity obscures the coupled processes that determine their electrochemical behavior. This mini review reframes MXene-based negative-electrode systems around the coupled logic of ion entry, charge compensation, and structural evolution. We discuss how interlayer galleries, surface terminations, confined water or solvent molecules, and metal-center redox cooperate or compete during charge storage. Particular emphasis is placed on the distinction between true intercalation, pseudo-intercalation, surface pseudocapacitance, partner-phase conversion/alloying in MXene-containing hybrid anodes, and electrolyte-regulated desolvation. Recent examples across alkali, aqueous zinc, and multivalent systems show that high-rate performance is obtained when ion access, electron transport, and lattice breathing are balanced rather than maximized independently. We highlight operando and multiscale measurements needed to connect local coordination changes with electrode-level kinetics, and we propose design principles for MXene-based negative-electrode systems that preserve redox accessibility while limiting restacking, oxidation, and parasitic interfacial reactions.

## 1 Introduction

MXenes entered electrochemical energy storage as a family of two-dimensional transition-metal carbides and nitrides whose formula, M_n+1_X_n_T_x_, immediately suggested a useful negative-electrode architecture: electronically conductive slabs separated by chemically addressable galleries (Naguib et al., 2011; Naguib and Gogotsi, 2015). Early work on Ti_3_C_2_T_x_ showed that cations can enter these galleries without the long diffusion distances typical of bulk insertion hosts, while surface terminations and interlayer species tune the local electrostatic environment (Come et al., 2015; Lukatskaya et al., 2013; Osti et al., 2016; Liu et al., 2026). This combination explains why MXenes often show sloping voltage profiles, high-rate capability, and partial pseudocapacitive behavior rather than the flat plateaus of a single-phase intercalation compound (Gao et al., 2020; Hart et al., 2019; Mu et al., 2019; Srimuk et al., 2016). For negative electrodes, the central question is therefore not only how much charge can be stored, but which atomic sites compensate that charge and how the layered framework responds.

**FIGURE 1:**
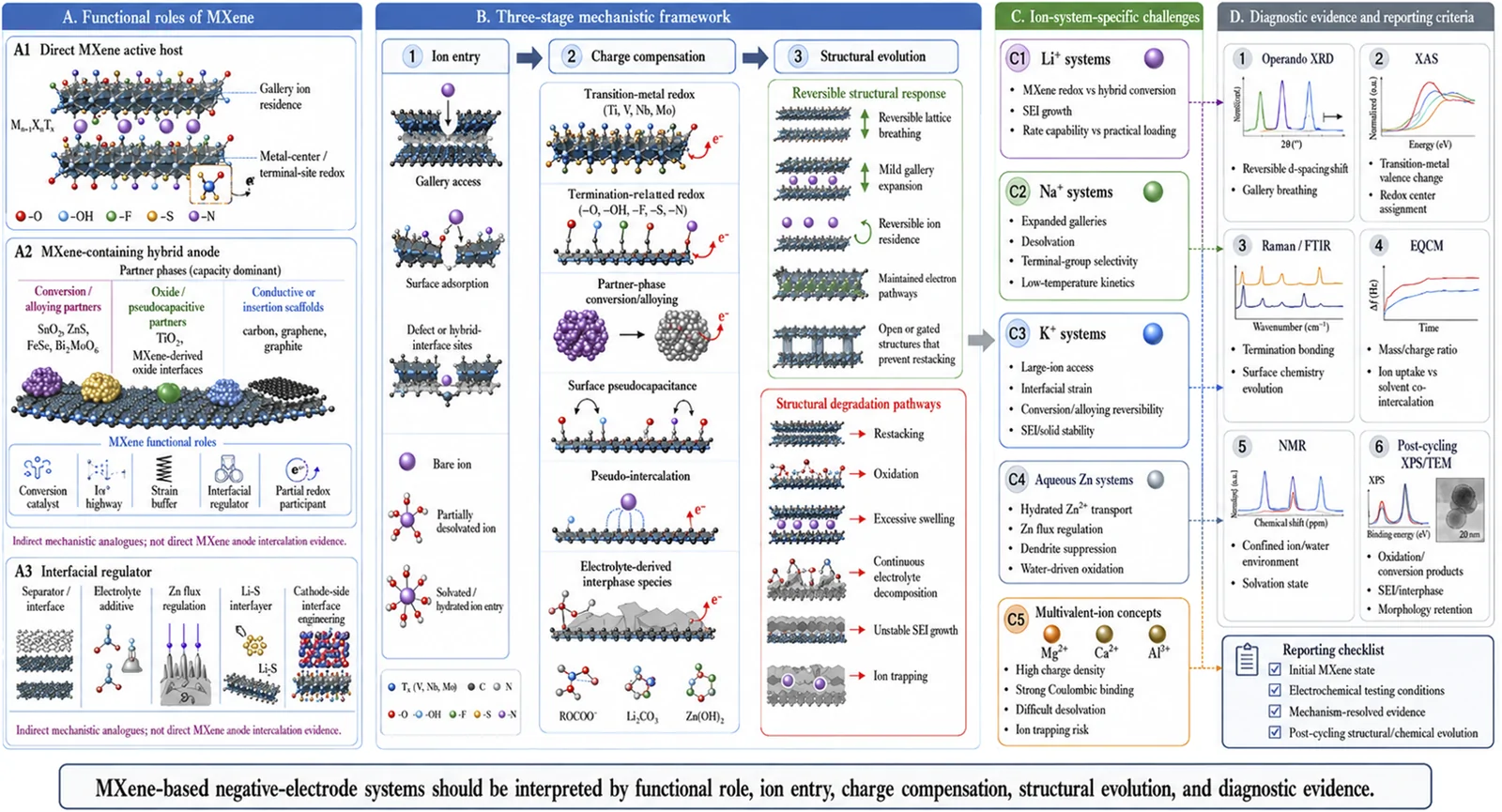
Mechanism-oriented framework for MXene-based negative-electrode systems. MXene-containing systems are classified according to their actual function, including direct MXene active hosts, MXene-containing hybrid anodes, and MXene-enabled interfacial regulators. Ion entry into MXene galleries, surfaces, defects, or hybrid interfaces is coupled with charge compensation at transition-metal centers, terminal groups, partner phases, or interphase species. These coupled processes drive reversible lattice breathing, gallery expansion, and ion residence, but may also trigger restacking, oxidation, unstable SEI growth, and parasitic interfacial reactions. The framework links Li^+^, Na^+^, K^+^, aqueous Zn systems, and broader multivalent-ion concepts with mechanism-resolved diagnostic evidence.

This mini review adopts a mechanism-first framework for MXene-based negative-electrode systems. The guiding sequence is ion entry, charge compensation, and structural evolution. Ion entry describes whether Li^+^, Na^+^, K^+^, Zn^2+^, Mg^2+^, or solvated complexes cross the surface and occupy interlayer, surface, defect, or hybrid-interface sites. Charge compensation asks whether electrons localize on Ti, V, Nb, Mo, guest metals, terminal O/F/OH groups, or coupled conversion products. Structural evolution then covers gallery expansion, termination rearrangement, oxidation, pillaring, aggregation, and heterostructure formation. Framing the literature in this order avoids a capacity-centered narrative and connects classical MXene chemistry with recent battery-oriented designs (Li et al., 2019; Maughan et al., 2021; Syamsai et al., 2021; Yang et al., 2016). Figure 1 illustrates this coupled mechanism map from MXene functional roles to ion entry, charge compensation, structural evolution, and diagnostic evidence. Table 1 summarizes ion-specific stress points in the three-stage framework, whereas Table 2 classifies MXene-based negative-electrode systems according to the actual function of MXene and the corresponding evidence status.

**TABLE 1:** Ion-specific stress points in the three-stage framework.

Ion system	Ion-entry challenge	Charge-compensation challenge	Structural-evolution challenge
Li^+^ systems	Distinguishing true gallery/interfacial ion uptake from SEI-related charge consumption and hybrid-particle reactions	Separating intrinsic MXene redox from partner-phase conversion/alloying and electrolyte-derived interphase reactions	Controlling SEI growth, maintaining scaffold conductivity, and preserving MXene/partner-phase contact during cycling
Na^+^ systems	Accommodating larger ions through expanded galleries, porous architectures, terminal-group selectivity, and partial desolvation	Distinguishing pseudo-intercalation and surface redox from simple adsorption or carbon/chalcogenide contribution	Maintaining reversible lattice breathing, pore retention, and low-temperature kinetic stability while suppressing restacking
K^+^ systems	Enabling large-ion access and desolvation through open heterostructures and conductive MXene-supported interfaces	Identifying the relative contribution of hybrid conversion/alloying partners versus MXene-mediated charge transfer	Buffering interfacial strain, limiting pulverization, and stabilizing conversion/alloying interfaces during repeated cycling
Aqueous Zn systems	Managing hydrated Zn^2+^ transport, Zn-ion flux, interfacial electric-field distribution, and water-mediated access to active interfaces	Distinguishing direct MXene charge storage from Zn plating/stripping regulation, oxide/MXene interfacial effects, or cathode-side reactions	Controlling water-mediated oxidation, swelling, dendrite growth, vacancy evolution, and interfacial degradation
Mg^2+^/Ca^2+^/Al^3+^ and broader multivalent-ion concepts	Overcoming strong solvation, high charge density, and difficult desolvation before ions can enter galleries or interfacial sites	Compensating high ionic charge without excessive Coulombic binding or irreversible trapping at terminal groups	Avoiding ion trapping, sluggish diffusion, surface-dominated storage, oxidation, and irreversible structural distortion

**TABLE 2:** Diagnostic map for MXene-based negative-electrode systems classified by MXene function and evidence status.

System or example type	Actual function of MXene	Ion-entry/interfacial process	Charge compensation/redox process	Structural evolution/main risk	Recommended diagnostic evidence	Evidence status
Ti_3_C_2_T_x_ films, MXene papers, pillared MXenes, MXene aerogels, and related MXene-only electrodes	Direct MXene active host	Ions enter interlayer galleries, surface sites, expanded/pillared channels, or confined hydrated environments	Charge is compensated mainly by transition-metal centers and terminal groups; pseudo-intercalation and surface redox may contribute	Reversible lattice breathing, gallery expansion, water redistribution, actuation, restacking, and oxidation may occur during cycling	Operando XRD for d-spacing changes; XAS/XPS for metal valence; EQCM/NMR for ion/solvent uptake; post-cycling XPS/TEM for oxidation and morphology	Direct MXene negative-electrode or direct MXene ion-storage evidence (Come et al., 2015; Gao et al., 2020; Lukatskaya et al., 2013; Maughan et al., 2020; Maughan et al., 2021; Mu et al., 2019; Osti et al., 2016; Song et al., 2022)
Nb-based MXenes, double-transition-metal MXenes, Mo_2_TiC_2_T_x_, and non-Ti MXene hosts	Direct MXene active host or composition-tuned MXene host	Ion entry occurs through MXene galleries or surface sites whose accessibility depends on M-site composition, gallery spacing, and terminal chemistry	Charge compensation is tuned by different transition-metal d states, metal-site redox accessibility, and termination-dependent electronic structure	Structural reversibility depends on termination stability, electrolyte compatibility, and resistance to oxidation or restacking	XAS for metal-specific redox; XRD for gallery evolution; XPS for terminal composition; electrochemical controls comparing different MXene compositions	Direct or composition-focused MXene host evidence (Bhat et al., 2025; Maughan et al., 2021; Syamsai et al., 2021; Yang et al., 2016)
SnO_2_/Ti_3_C_2_T_x_, ZnS/Ti_3_C_2_T_x_, Bi_2_MoO_6_/MXene, FeSe/MXene, and transition-metal chalcogenide–MXene hybrids	MXene-containing hybrid anode scaffold and possible partial redox participant	Ions access MXene-supported hybrid interfaces and active nanoparticles; MXene galleries may also contribute limited ion transport	Partner phases usually dominate conversion/alloying or major faradaic capacity, while MXene provides conductive coupling, polar surfaces, ion pathways, and possible surface redox	MXene buffers volume change, suppresses aggregation, improves electron transport, and stabilizes interfaces; risks include continuous SEI growth and partner-phase reconstruction	Control electrodes without MXene or without the partner phase; operando/*ex situ* XRD for conversion products; XPS/XAS for component-specific redox; TEM for particle reconstruction; SEI analysis	Direct hybrid-anode evidence, but not direct proof of intrinsic MXene intercalation (Cao et al., 2021a; Cao et al., 2021b; Liu et al., 2019; Rehman et al., 2024; Yang et al., 2022; Zhang et al., 2019)
Carbon/MXene, graphene/MXene, graphite/MXene, polydopamine/MXene, and MXene–carbonaceous architectures	Conductive scaffold, insertion scaffold, or interfacial kinetic regulator	Ion transport occurs through carbon/MXene interfaces, porous conductive networks, or graphite-related insertion pathways	Carbon or graphite may contribute insertion/storage, while MXene improves conductivity, polarity, interfacial contact, and charge-transfer kinetics	Improved percolation, reduced polarization, better mechanical integrity, and suppressed interfacial degradation may occur; the MXene contribution must be separated from carbon/graphite storage	Control electrodes separating MXene and carbon/graphite contributions; GITT/EIS for transport kinetics; post-cycling XPS/TEM for interface stability; practical loading and areal-capacity reporting	Hybrid scaffold or insertion-interface evidence; not direct evidence of intrinsic MXene intercalation unless MXene redox is independently verified (Chen et al., 2025; Feng et al., 2023; Li et al., 2020)
Aqueous Zn-ion capacitors or aqueous cells using MXene as a negative electrode	Direct aqueous negative-electrode host or MXene-based device electrode	Hydrated ions and confined water interact with MXene galleries, surfaces, or pillared structures	Charge storage may involve surface redox, pseudo-intercalation, capacitive behavior, or hybrid-device charge balance depending on the electrode architecture	Water-mediated swelling, gallery hydration, oxidation, conductivity loss, and structural retention determine reversibility	Hydration-state tracking; operando XRD; EQCM/NMR for ion/water uptake; XPS for oxidation; conductivity and phase-purity checks after cycling	Direct aqueous negative-electrode or MXene-based device evidence (Maughan et al., 2020; Wang et al., 2019)
Aqueous Zn systems using MXene as an electrolyte additive, separator modifier, protective interlayer, or Zn-deposition regulator	MXene-enabled interfacial regulator	MXene regulates hydrated Zn^2+^ flux, local electric fields, deposition morphology, and separator/electrolyte behavior rather than serving as the main negative-electrode host	Charge transfer is mainly associated with Zn plating/stripping, Zn-interface regulation, or oxide/MXene interfacial effects, not direct MXene gallery intercalation	Dendrite suppression, water-activity modulation, vacancy stabilization, interfacial stabilization, and MXene oxidation control are central issues	Zn deposition morphology; symmetric-cell tests; HER/oxidation analysis; XPS/TEM after cycling; water-content and electrolyte-control experiments	Interfacial evidence; indirect for direct MXene-host intercalation (Fang et al., 2025; Sun et al., 2021; Yan et al., 2024)
Li–S interlayers, separator coatings, cathode-side MXene interfaces, and lithium-plating regulation layers	MXene-enabled interfacial regulator or indirect mechanistic analogue	MXene surfaces interact with polysulfides, solvated intermediates, lithium-plating interfaces, or separator/interlayer environments	Surface-mediated catalysis, polar-site adsorption, and interfacial charge transfer may occur, but these processes are not direct MXene-host intercalation in direct MXene anodes	Stabilized interphase, suppressed shuttle effect, improved sulfur conversion, or guided lithium plating may be observed	Polysulfide adsorption/conversion tests; Li plating morphology; separator/interlayer stability; XPS for interphase chemistry; electrochemical controls without MXene	Indirect mechanistic analogue; not direct evidence of MXene active-host behavior (Deng et al., 2024; Sandhu et al., 2024; Wang et al., 2021; Wu et al., 2025)
Mg^2+^, Ca^2+^, Al^3+^, and broader multivalent-ion MXene concepts	Emerging MXene host or interfacial system	High-charge-density ions require pre-expanded galleries, weakly coordinating terminations, partial desolvation, or transport as solvated complexes	Host metal centers and terminal groups must compensate high ionic charge without trapping ions too strongly	Strong Coulombic binding, slow diffusion, ion trapping, oxidation, and surface-dominated storage are major risks	Solvation/desolvation analysis; bare-ion versus solvated-ion calculations; operando XRD/XAS; NMR or MD simulations of solvation; post-cycling phase and valence analysis	Emerging or conceptual evidence; requires stronger operando validation (Bhat et al., 2025)

The scope is intentionally narrower than that of a general review of MXene energy storage. We focus on negative-electrode processes in rechargeable batteries and battery-like hybrid devices because this is where the language of intercalation is most often used loosely. In the literature, an MXene-based negative-electrode system may refer to a nearly pristine Ti_3_C_2_T_x_ film, a delaminated or pillared direct MXene host, a three-dimensional aerogel, or an MXene-containing hybrid anode in which MXene is only one component of a conversion or alloying electrode. These systems should not be assigned the same mechanistic label. A sloping galvanostatic profile, a high capacitive contribution from scan-rate analysis, or a small lattice-spacing change does not by itself prove reversible intercalation. Conversely, a hybrid electrode can still illuminate MXene chemistry if it reveals how terminal groups, confined solvent, or electronic coupling alter ion transfer. The following sections therefore read the evidence by function rather than by material class. Unlike performance-centered reviews that catalogue MXene-based electrodes by battery chemistry, this mini review uses intercalation validity, charge-compensation sites, and structural reversibility as the primary criteria for evaluating MXene-based negative-electrode mechanisms.

We use “MXene-based negative-electrode systems” as an umbrella term, but distinguish three functional roles throughout the review. “Direct MXene anodes” refer to systems in which ion residence, charge compensation, and structural response occur mainly within MXene galleries, surface redox sites, or transition-metal centers. “MXene-containing hybrid anodes” refer to composite negative electrodes in which MXene provides conductive pathways, ion-accessible interfaces, strain buffering, catalytic interfaces, or partial redox participation, while partner phases may dominate conversion, alloying, or other major faradaic reactions. “MXene-enabled interfacial regulators” refer to separators, interlayers, electrolyte additives, Zn-deposition modifiers, Li–S interfacial layers, or related interface-control components. These interfacial examples are discussed as indirect mechanistic analogues rather than as direct evidence of MXene-host intercalation.

The proposed framework also clarifies how Tables 1, 2 should be read. Table 1 is not a performance ranking; it summarizes ion-specific stress points in the three-stage framework. Table 2 further classifies MXene-based negative-electrode systems according to the actual function of MXene and the corresponding evidence status. A lithium hybrid electrode and an aqueous zinc interfacial system may both fall under the umbrella of MXene-based negative-electrode systems, yet their MXene functions, evidence status, and limiting steps differ. In one case, the critical issue may be solid-electrolyte interphase formation on a low-potential conductive scaffold; in the other, it may be hydrated-ion transport and water-driven oxidation. This comparison turns scattered reports into testable questions. When a new MXene-based negative-electrode system is proposed, the first evaluation should ask which functional category and mechanistic row it resembles and what evidence would falsify that assignment.

## 2 Interlayer galleries as ion-selective reaction spaces

The first mechanistic level is the interlayer gallery. In Ti_3_C_2_T_x_ films, cation insertion can occur with subtle but measurable lattice breathing, water redistribution, and changes in local coordination. *In situ* and multiscale studies show that ions do not simply fill empty space; they alter hydration, screen surface charge, and modify the mechanical state of stacked sheets (Come et al., 2015; Gao et al., 2020). The gallery is therefore both a transport channel and a reaction space. Small or weakly solvated ions can approach terminal oxygen sites closely, whereas larger alkali ions or hydrated multivalent ions may require expanded spacing, preinserted spacers, or partial desolvation. These constraints explain why a material that performs well for Li^+^ does not necessarily translate directly to Na^+^, K^+^, or Zn^2+^ storage.

Interlayer engineering aims to separate useful swelling from destructive restacking. Pillared and porous MXenes increase gallery accessibility, shorten ion pathways, and stabilize the stacked structure during cycling (Handoko et al., 2019; Mathew et al., 2022; Maughan et al., 2020; Maughan et al., 2021). Aerogel and three-dimensional MXene assemblies similarly convert dense lamellae into connected ion/electron networks, improving high-mass-loading sodium storage and aqueous hybrid capacitors (Song et al., 2022; Wang et al., 2019). However, wider spacing is not automatically beneficial. If pillars are redox-inactive, block conductive contact, or promote excessive electrolyte co-intercalation, they may dilute volumetric capacity or accelerate side reactions. Rational design should therefore specify which ion species enters the gallery, how much of the solvation shell is retained, and which host atoms accept the compensating electrons.

Recent work illustrates this point most clearly for desolvation and confined solvent effects. Ion desolvation in Ti_3_C_2_ MXene electrodes can boost charge storage when the interfacial environment lowers the penalty for bringing ions into redox-active proximity (Bo et al., 2025). Conversely, interlayer water can facilitate diffusion by screening electrostatic interactions, but it can also hinder storage by occupying sites and promoting oxidation (Li et al., 2019; Osti et al., 2016). The same water-mediated logic extends to aqueous zinc-ion systems, where MXene-containing interfaces guide Zn deposition, suppress parasitic reactions, or stabilize iodine conversion (Fang et al., 2025; Sun et al., 2021; Yan et al., 2024). These examples show that the electrolyte is part of the negative-electrode and interfacial mechanism, not a passive ion reservoir.

The gallery should also be viewed as a mechanically active space. Electrochemical actuation experiments showed that cation insertion can bend or expand MXene papers, indicating that ion uptake is coupled to stress even when the host appears macroscopically stable (Come et al., 2015). During battery cycling, this stress is distributed across flakes, interflake contacts, binders, and pores. Dense films may retain excellent electronic conductivity but lose ion accessibility as cycling compacts the stack. Highly porous networks may admit ions rapidly but suffer from lower tap density or greater exposed surface area for parasitic reactions. The most useful architectures sit between these extremes: they maintain percolated electron pathways while leaving enough free volume for reversible solvation changes and lattice breathing. This balance explains why many recent MXene-containing negative-electrode designs use carbon spacers, molecular pillars, or oxide/chalcogenide particles as structural regulators rather than simple capacity additives (Fang et al., 2020; Li et al., 2020; Liu et al., 2019; Mathew et al., 2022; Maughan et al., 2020; Zhang et al., 2019).

A second implication is that intercalation selectivity is chemical, not merely geometric. Terminal O and OH groups often provide stronger alkali-ion binding, whereas F-rich surfaces may, in some cases, reduce ion affinity, alter wettability, or decrease the density of redox-accessible oxygen-containing sites. Intercalated water and organic molecules modify these interactions by changing local dielectric screening. In some cases, preinserted ions or molecules create a more open gallery and accelerate the target ion; in others, they compete for adsorption sites or form kinetically trapped complexes. A useful report of MXene-based negative-electrode performance should therefore specify the synthesis route, delamination agent, terminal composition, preintercalated species, drying history, and electrolyte water content. These details are often treated as materials-processing variables, but in MXenes they define the initial state of the electrochemical reaction.

This initial-state sensitivity partly explains discrepancies among reports that use nominally similar Ti_3_C_2_T_x_. Different etching and delamination routes produce different flake sizes, defect densities, residual Al contents, terminal ratios, and intercalated species. Drying can collapse galleries, while storage in water can promote oxidation. Electrode fabrication adds further variables: polymer binders may block galleries, carbon additives may change percolation, and calendaring may reduce porosity. Mechanistic comparison across studies therefore requires more than matching the MXene formula. It also requires matching the chemical and morphological state of the electrode before cycling. For a mini review, this is why synthetic history is treated as part of the mechanism rather than as background preparation detail.

## 3 Charge compensation: Metal centers, terminations, and pseudo-intercalation

After ion entry, the key question is where the electron goes. MXene slabs contain transition-metal centers whose d states are sensitive to termination, composition, and intercalated guests. Experiments and theory show that termination and intercalation can shift electronic properties, changing work function, conductivity, and redox accessibility (Handoko et al., 2019; Hart et al., 2019; Wang et al., 2015). In acidic or aqueous electrolytes, proton or metal-cation insertion may appear capacitive at the electrode scale but still involve local redox of Ti or other metal centers, hence the term pseudo-intercalation (Mu et al., 2019). This behavior differs from double-layer capacitance because charge is not stored only by electrostatic separation; it also involves partial changes in metal oxidation state and terminal bonding.

Surface terminations are not spectators. O, OH, F, Cl, S, and N-containing terminals alter ion adsorption energies, redox potentials, diffusion barriers, hydrophilicity, and resistance to oxidation. Nitrogen-terminal engineering, for example, enables fast sodium storage at low temperature by improving Na^+^ interaction and kinetics (Xia et al., 2022). Sulfurized or chalcogen-containing MXene hybrids often add conversion-type redox while retaining a conductive MXene scaffold (Cao B. et al., 2021; Wang et al., 2021; Yang et al., 2022; Zhang et al., 2019). The challenge is to distinguish terminal-group redox from redox of the transition-metal layer and from reactions of attached nanoparticles. Without operando spectroscopy or careful *ex situ* controls, a high pseudocapacitive fraction can be overinterpreted as intrinsic MXene behavior.

Compositional tuning expands the charge-compensation map. Nb-based MXenes, double-transition-metal MXenes, Mo_2_TiC_2_, and non-Ti MXenes offer different d-band positions and ion-binding strengths (Bhat et al., 2025; Maughan et al., 2021; Syamsai et al., 2021; Yang et al., 2016). These changes can increase theoretical capacity or improve rate performance, but they also alter termination stability and electrolyte compatibility. Recent MXene-containing hybrid anodes and interfacial architectures further complicate the picture by coupling MXenes with SnO_2_, ZnS, FeSe, Bi_2_MoO_6_, TiO_2_, carbon, phosphorene, or sulfur hosts (Cao J. et al., 2021; Deng et al., 2024; Fang et al., 2020; Li et al., 2020; Li et al., 2024; Liu et al., 2019; Zhang et al., 2019). In such systems, MXene may serve simultaneously as conductive matrix, strain buffer, ion highway, catalytic interface, and sometimes a redox participant. Mechanistic claims should therefore identify which component stores charge and which component mainly modulates kinetics.

This ambiguity is most evident when b-value analysis or Dunn-type separation is used to claim pseudocapacitive storage. These methods are useful for comparing kinetics, but they partition current by time scale rather than by atomic mechanism. A fast conversion reaction on nanosized SnO_2_, a surface-confined redox process on Ti sites, and ion adsorption at defective carbon can all contribute to a current component that scales nearly linearly with scan rate. Assigning all such current to MXene pseudocapacitance overstates the intrinsic role of the host. Stronger evidence comes from correlated measurements: shifts in Ti, V, Nb, or Mo absorption edges; reversible changes in terminal-group vibrational modes; mass changes from quartz-crystal microbalance; and operando diffraction that tracks gallery spacing. The most convincing papers combine at least two of these signals with electrochemical controls that isolate the partner phase (Gao et al., 2020; Mu et al., 2019; Osti et al., 2016; Wang et al., 2015). For example, operando XRD primarily identifies gallery breathing, XAS tracks transition-metal valence changes, Raman or FTIR probes termination bonding, EQCM distinguishes ion uptake from solvent co-intercalation, and post-cycling XPS/TEM can reveal oxidation or conversion products. Only by combining these signals can pseudo-intercalation be separated from surface adsorption or partner-phase conversion.

Termination chemistry also creates a trade-off between activity and durability. Oxygen-rich surfaces usually favor stronger cation binding and greater redox participation, but they may also promote irreversible oxidation in water-containing electrolytes. Fluorine termination can improve chemical stability in some environments, although it may lower conductivity or reduce ion affinity. Sulfur, nitrogen, or halogen engineering can tune adsorption and electronic structure, yet the introduced groups may be labile under deep reduction. Terminal-group optimization should therefore be evaluated over the intended voltage range, not only by initial adsorption calculations. For MXene-based negative-electrode systems, the relevant question is whether the termination remains available and electronically coupled after solid-electrolyte interphase formation, repeated ion insertion, and exposure to trace water or oxygen.

A practical consequence is that mechanistic language should remain conservative and internally consistent. In this review, “intercalation” is reserved for reversible ion residence within MXene galleries or crystallographic spaces that remain substantially intact during cycling. “Pseudo-intercalation” denotes fast insertion or near-surface ion residence accompanied by local redox of transition-metal centers or terminal groups, but without a classical two-phase plateau. “Surface pseudocapacitance” refers to redox-confined surface or near-surface sites rather than purely electrostatic double-layer charging. “Conversion” and “alloying” are used only when partner phases form new compounds or alloys during cycling, and should not be assigned to MXene itself unless direct phase and redox evidence is provided. Separators, interlayers, electrolyte additives, Zn-deposition modifiers, and Li–S interfacial layers are described as “MXene-enabled interfacial regulators” or “indirect mechanistic analogues,” not as direct evidence of MXene-host intercalation. This vocabulary is not semantic housekeeping; it determines which degradation pathways should be expected and which design lever should be changed next.

The preceding discussion defines how ion entry, charge compensation, and structural evolution should be separated when interpreting MXene electrochemistry. These criteria become more discriminating when different carrier ions are compared. Table 1 summarizes the ion-specific stress points that guide the following discussion. Li^+^ storage often tests whether MXene redox can be separated from hybrid conversion and SEI growth, whereas Na^+^ and K^+^ storage impose stronger constraints on gallery accessibility, desolvation, and strain accommodation. Aqueous Zn systems and broader multivalent-ion concepts further emphasize hydration, charge density, and interfacial regulation. The following sections therefore apply the same three-stage framework to representative ion systems rather than treating each battery chemistry as an independent material category.

## 4 Cross-cutting electrolyte and interphase effects

Electrolyte chemistry cuts across all three stages of the proposed framework because the ion that reaches MXene is rarely a bare ion moving through an inert medium. Solvation and desolvation first determine whether the carrier ion can enter an interlayer gallery, adsorb on a terminal group, or approach a hybrid interface. For Li^+^, Na^+^, and K^+^ systems, the apparent charge-transfer resistance may include the energetic cost of removing solvent molecules before surface adsorption or gallery entry. For aqueous Zn systems and broader multivalent-ion concepts, hydrated or strongly solvated ions can be too large or too strongly coordinated to behave like the nominal bare ions used in simplified structural models. Confined water can lower migration barriers by screening electrostatic interactions, but it can also occupy active sites, promote co-intercalation, and accelerate oxidation. Thus, gallery spacing should be evaluated together with solvation structure, electrolyte composition, and water content rather than treated as a purely geometric descriptor (Bo et al., 2025; Li et al., 2019; Osti et al., 2016).

Electrolyte-mediated screening also affects charge compensation. Terminal groups such as–O, –OH, –F, –S, and–N do not interact with ions in isolation; their effective binding strength and redox accessibility depend on local dielectric environment, proton activity, salt concentration, and interfacial solvent organization. A polar or hydrated interface may facilitate ion approach and local electron transfer, whereas excessive solvent retention may separate ions from redox-active metal centers or promote side reactions. In hybrid electrodes, electrolyte composition further influences whether the measured current mainly reflects MXene surface redox, partner-phase conversion/alloying, carbon adsorption, or electrolyte-derived interphase reactions. For this reason, electrochemical signatures such as sloping voltage profiles, high capacitive fractions, or low impedance should not be assigned to intrinsic MXene pseudocapacitance without complementary evidence of metal-center redox, terminal-group evolution, or partner-phase controls (Hart et al., 2019; Mu et al., 2019; Wang et al., 2015).

Electrolyte decomposition and water activity finally determine whether structural evolution remains reversible. In nonaqueous alkali-ion systems, unstable SEI growth can consume electrolyte, mask MXene redox sites, increase interflake resistance, or obscure the contribution of hybrid partner phases. In aqueous systems, dissolved oxygen, pH, water activity, and potential window can drive MXene oxidation, hydrogen evolution, swelling, or oxide/MXene interface formation. Some electrolyte-driven transformations may be useful when they create stable oxide interfaces, suppress dendrites, or improve ion-flux regulation, but they should not be described as pristine MXene intercalation. Future studies should therefore pair electrolyte design with post-cycling analysis of terminal composition, oxidation state, SEI/interphase chemistry, and gallery structure. This treatment makes electrolyte and interphase evolution a mechanistic link between ion entry, charge compensation, and structural reversibility rather than a background testing condition (Fang et al., 2025; Li et al., 2019; Sun et al., 2021).

## 5 Alkali-ion negative-electrode systems: Size, solvation, and hybrid redox

Lithium-ion storage in MXene-based negative-electrode systems benefits from the small size of Li^+^ and the high conductivity of MXene sheets, yet direct MXene anodes such as pristine Ti_3_C_2_T_x_ often deliver lower reversible capacity than conversion or alloying hosts. Consequently, many MXene-containing lithium hybrid anodes use MXene as a conductive scaffold, ion-accessible interface, and strain-buffering matrix for nanoparticles or molecularly connected carbon phases. SnO_2_/Ti_3_C_2_T_x_, ordered mesoporous polydopamine/MXene, Bi_2_MoO_6_/MXene, ZnS/Ti_3_C_2_T_x_, and MXene-configured graphite illustrate this strategy (Cao B. et al., 2021; Chen et al., 2025; Li et al., 2020; Liu et al., 2019; Zhang et al., 2019). Their common mechanism is not simple intercalation. Instead, it is a division of labor in which MXene preserves electrical continuity, exposes polar surfaces, and buffers volume changes, while the partner phase supplies alloying, conversion, or interphase stabilization.

Sodium and potassium storage emphasize ion size and structural breathing. The larger Na^+^ and K^+^ ions make dense restacked films kinetically unfavorable, so porous MXene aerogels, pillared MXenes, carbon-coated MXene/selenide structures, and chalcogenide-MXene-carbon heterostructures have been developed to maintain open channels and fast electron transport (Cao J. et al., 2021; Maughan et al., 2020; Song et al., 2022; Yang et al., 2022). The strongest recent sodium examples combine interlayer spacing, surface chemistry, and mechanical accommodation rather than relying on any single parameter. For potassium, the large ionic radius makes stable interfaces and strain buffering especially important. MXene-containing FeSe and layered heterostructures illustrate how a conductive, flexible scaffold can make conversion or alloying chemistry more reversible (Cao J. et al., 2021; Yang et al., 2022).

Lithium–sulfur and hybrid-battery studies are not treated here as direct evidence of MXene-host intercalation in direct MXene anodes. Instead, they are used as interfacial analogues that reveal how MXene surfaces regulate solvated intermediates, lithium plating, interphase formation, and surface-mediated conversion kinetics. MXene surfaces can adsorb polysulfides, catalyze conversion, guide lithium plating, and stabilize separators or interlayers (Deng et al., 2024; Li et al., 2024; Sandhu et al., 2024; Wang et al., 2021; Wu et al., 2025). These functions show that redox kinetics often depend on interfacial orbital interactions and polar surface sites. For MXene-based negative-electrode systems, the same principle suggests that MXene surfaces should be designed to manage solvated intermediates and interphase formation, not only to expose more area. Otherwise, high initial capacity may be accompanied by continuous electrolyte decomposition or unstable solid-electrolyte interphase growth.

For Li^+^ systems, the most direct MXene contribution is often rate capability rather than maximum capacity. The metallic conductivity of Ti_3_C_2_T_x_ and related phases can lower electrode polarization, while polar surfaces can improve contact with metal oxides, sulfides, or carbon. Yet the same surface polarity can increase electrolyte decomposition if the anode operates at low potential. A design that appears excellent at low mass loading may lose its advantage when the electrode is thickened, because electrolyte consumption, tortuosity, and interflake resistance become more important. It is therefore essential to report areal capacity, electrode density, and post-cycling surface chemistry when evaluating whether MXene functions as a practical negative-electrode component or merely as a nanoscale conductive additive.

For Na^+^ and K^+^ systems, desolvation and interphase chemistry deserve the same attention as bulk diffusion. The larger ionic radii of Na^+^ and K^+^ make the first contact with terminal groups more selective, and the energy cost of shedding solvent can dominate the apparent charge-transfer resistance. Nitrogen terminals, carbon interlayers, and MXene-carbonaceous nanoribbons can be read as attempts to smooth this interfacial transition (Cao J. et al., 2021; Feng et al., 2023; Xia et al., 2022). In this sense, the best alkali-ion MXene-based negative-electrode systems are not simply open structures; they are interfaces that choreograph desolvation, adsorption, electron transfer, and strain relaxation. This perspective helps explain why some composites outperform pristine MXenes even when their theoretical capacity is not substantially higher.

Thus, the dominant challenge shifts across alkali-ion systems: Li^+^ storage is often limited by separating MXene redox from hybrid conversion and SEI growth; Na^+^ storage is governed by gallery accessibility, desolvation, and terminal chemistry; K^+^ storage is constrained more strongly by interfacial strain, large-ion transport, and the reversibility of conversion/alloying partners.

## 6 Aqueous Zn chemistry and broader multivalent-ion concepts

Aqueous batteries highlight the dual role of water. Water improves ionic conductivity and can lower barriers for proton or metal-ion migration, yet it also changes MXene oxidation, termination exchange, and hydrogen evolution. Direct MXene negative electrodes in aqueous zinc-ion capacitors demonstrate how a conductive, flexible negative electrode can pair with pseudocapacitive or conversion-type positive electrodes (Maughan et al., 2020; Wang et al., 2019). More recent zinc-related work uses Ti_3_C_2_T_x_ as an electrolyte additive, separator modifier, protective interlayer, or interfacial regulator to guide Zn^2+^ flux and reduce dendrites (Fang et al., 2025; Sun et al., 2021; Yan et al., 2024). In these cases, MXene should not be interpreted as the main negative-electrode host. Rather, it functions as an interfacial regulator that controls Zn-ion flux, local electric fields, deposition morphology, or separator/electrolyte behavior. These studies are valuable for MXene-based negative-electrode systems because they show how charged surfaces and hydration shells regulate metal-ion deposition and stripping.

Multivalent ions provide a stronger test of the intercalation-charge compensation framework. Mg^2+^, Al^3+^, Ca^2+^, and Zn^2+^ carry more charge per ion, but stronger electrostatic interactions and larger hydrated or solvated structures can slow migration through narrow galleries. Studies on direct MXene negative electrodes and MXene-containing interfacial systems illustrate the importance of host structure and interlayer chemistry, while a recent review of multivalent MXene batteries suggests that high-charge-density ions require pre-expanded galleries, weakly coordinating terminations, or partial desolvation before insertion (Bhat et al., 2025). When these conditions are not met, apparent capacity may arise from surface reactions, electrolyte decomposition, or conversion of hybrid phases rather than reversible multivalent intercalation. Mechanistic assignments should therefore pair electrochemical data with structural probes and oxidation-state analysis.

Recent zinc-interface and lithium-electrode examples further shift attention from capacity to reversibility, interfacial stabilization, and evidence-based role assignment. Orbital hybridization at MXene-oxide interfaces can stabilize oxygen vacancies and improve aqueous zinc-ion reversibility (Fang et al., 2025). MXene-containing polymer or graphite architectures can improve ion transport and interface stability under harsh conditions (Chen et al., 2025; Wu et al., 2025). Yet these successes are highly interfacial: MXene may not be the dominant capacity carrier, but it controls charge transfer, local electric fields, or intermediate adsorption. This distinction is important for miniaturized and flexible devices, where high volumetric loading and mechanical integrity may matter as much as gravimetric capacity (Feng et al., 2023; Wang et al., 2019).

In aqueous cells, MXene oxidation is both a failure mode and a possible design tool. Mild oxidation can create oxide/MXene heterointerfaces, vacancies, or porous frameworks that improve adsorption and ion transport, whereas uncontrolled oxidation consumes conductive MXene and changes the voltage response. The difference between these outcomes depends on pH, dissolved oxygen, potential window, terminal groups, and electrolyte additives. Reports of MXene-based aqueous negative-electrode systems should therefore track not only capacity retention but also phase purity and conductivity after cycling. When an oxide layer is intentionally generated, the mechanism should be described as an MXene-derived or MXene-oxide interface rather than as pristine MXene intercalation.

Multivalent systems further emphasize charge density. In principle, a divalent ion can deliver twice the charge per insertion event, but the host must compensate that charge without trapping the ion too strongly. Strong binding can flatten diffusion pathways, distort terminal groups, or lock hydrated ions at gallery entrances. This is why theoretical studies of Ca-ion or Al-ion MXenes must be interpreted together with realistic electrolyte chemistry. If a calculation assumes a bare ion but the experiment transports a solvated complex, the predicted diffusion barrier may not describe the operating cell. Future multivalent work should therefore integrate molecular dynamics of solvation, density-functional calculations of surface binding, and operando structural measurements under aqueous or nonaqueous conditions.

## 7 Mechanism-resolved characterization: Distinguishing intercalation, adsorption, and conversion

A mechanism-first framework requires characterization signals that can separate true intercalation from surface adsorption, interfacial reactions, and partner-phase conversion. No single method is sufficient, because sloping voltage profiles, high capacitive fractions, or low impedance can arise from several atomic processes. Operando and post-cycling measurements should therefore be interpreted together: structural probes identify gallery breathing, spectroscopies assign redox centers and terminal-group changes, mass-sensitive methods distinguish ion uptake from solvent co-intercalation, and microscopy or surface analysis reveals irreversible oxidation, SEI growth, or partner-phase reconstruction. Table 3 summarizes practical diagnostic signals for assigning MXene-based negative-electrode mechanisms.

**TABLE 3:** Mechanism-resolved characterization signals for distinguishing intercalation, adsorption, interfacial reactions, and conversion.

Technique	Signal supporting intercalation or pseudo-intercalation	Signal suggesting adsorption, interfacial reaction, or conversion
Operando XRD	Reversible d-spacing shift synchronized with charge/discharge; reproducible gallery breathing without phase collapse	No gallery change despite large capacity; irreversible peak shift or broadening; new crystalline phases from conversion products
XAS	Reversible Ti, V, Nb, or Mo edge shift correlated with electrochemical state; recoverable local coordination changes	Dominant edge changes from partner phases; irreversible oxidation of MXene; weak or absent MXene metal-center redox despite high capacity
Raman/FTIR	Reversible changes in terminal-group vibration or bonding environment during cycling	New SEI, adsorbate, or electrolyte-decomposition bands; irreversible loss or rearrangement of terminal groups
EQCM	Mass/charge ratio consistent with ion uptake or defined solvated-ion uptake; reversible mass change over cycling	Excess mass gain from solvent co-intercalation, salt accumulation, SEI growth, or electrolyte decomposition
NMR	Confined ion/water environments, reversible mobility changes, or distinguishable solvated/intercalated species	Trapped solvent, irreversible electrolyte products, or persistent bound species after discharge
Post-cycling XPS/TEM	Retained MXene phase, preserved layered morphology, and reversible or limited surface-state changes	Oxide growth, unstable SEI, conversion products, nanoparticle reconstruction, particle pulverization, or severe restacking
Control electrodes	Comparable MXene-only, partner-only, and hybrid electrodes clarify whether MXene stores charge or mainly improves kinetics	Capacity dominated by partner phases, carbon, electrolyte-derived interphase, or separator/interlayer effects rather than intrinsic MXene redox

These criteria should be used cautiously rather than as isolated proof. For example, a reversible d-spacing change supports gallery participation but does not by itself identify the redox center. Similarly, a high pseudocapacitive fraction from scan-rate analysis indicates fast kinetics but cannot distinguish MXene surface redox from nanosized conversion, carbon adsorption, or electrolyte-derived processes. The strongest assignments require at least one structural signal, one redox or chemical-state signal, and one control experiment that separates direct MXene storage from hybrid or interfacial contributions.

## 8 Outlook: Experiments and design rules

A mechanism-centered review suggests four design rules. First, tune the gallery for the actual mobile species, including its solvation shell, rather than for a nominal bare ion radius. Second, select terminations that balance ion affinity, electronic conductivity, redox potential, and oxidation resistance. Third, separate the roles of MXene and partner phases in hybrids: conductive scaffold, ion sieve, catalytic surface, strain buffer, and active redox host should be assigned with evidence. Fourth, preserve structural reversibility under realistic loading. Open, pillared, or porous structures are useful only if they remain connected and chemically stable over long cycling (Fang et al., 2020; Kalsoom et al., 2025; Mathew et al., 2022; Rehman et al., 2024). At the device level, charging protocols should also be matched with MXene-mediated ion transport and interphase stability, because excessive overpotential, low-temperature operation, or fast charging at high state of charge may amplify desolvation barriers, SEI growth, and structural stress.

Building on the diagnostic criteria summarized in Section 7 and Table 3, future studies should combine mechanism-resolved characterization with practical electrode-level reporting. Kinetic analysis should be reported together with electrode thickness, mass loading, electrolyte composition, and voltage window; otherwise, pseudocapacitive fractions derived from scan-rate fitting can be misleading.

Three open questions are especially important for a mini review agenda. First, what fraction of charge in a given direct MXene anode or MXene-containing hybrid anode is compensated by transition-metal redox rather than by double-layer charging or partner-phase conversion? Second, how do terminal groups evolve under realistic cycling, especially after the first formation cycles when the electrolyte-derived interphase is established? Third, can galleries be designed to favor selective desolvation and reversible ion residence without sacrificing volumetric density? Answering these questions will require both model electrodes and practical composites. Model films provide clean spectroscopic signatures, whereas practical porous or hybrid electrodes reveal how those signatures survive under high loading.

At minimum, future reports should disclose four groups of information: the initial MXene state, electrochemical testing conditions, mechanism-resolved evidence, and post-cycling structural/chemical evolution. The initial-state description should include termination composition, interlayer species, flake size, defect density, and drying or storage history. Testing conditions should include electrode loading, voltage window, electrolyte formulation, and ion-solvation environment. Mechanistic evidence should include operando structural tracking, redox-state analysis, and controls that separate MXene from partner-phase contributions, followed by post-cycling analysis of phase, valence, interphase, and morphology evolution. Such reporting would make it possible to compare MXene-based negative-electrode systems across different ions without reducing their behavior to capacity and rate capability alone.

A final design implication is that MXene-based negative-electrode systems should be optimized as coupled electrochemical systems. The MXene flake, termination layer, interlayer water, electrolyte salt, binder, conductive network, and partner active material all influence the same redox event. Treating one component as a passive support can obscure why a material succeeds or fails. The most convincing future studies will report a mechanism diagram that connects ion path, electron path, redox center, structural response, and degradation route. This level of evidence is needed for MXene-based negative-electrode systems to progress from attractive high-rate components to predictable negative-electrode platforms for rechargeable batteries.
